# CIF (Crystallographic Information File): A Standard for Crystallographic Data Interchange

**DOI:** 10.6028/jres.101.035

**Published:** 1996

**Authors:** I. D. Brown

**Affiliations:** Brockhouse Institute for Materials Research, McMaster University, Hamilton, Ontario, Canada

**Keywords:** crystallographic information, file structures, relational databases, STAR files

## Abstract

The Crystallographic Information File (CIF) uses the self-defining STAR file structure. This requires the creation of a dictionary of data names and definitions. A basic dictionary of terms needed to describe the crystal structures of small molecules was approved in 1991 and is currently used for the submission of papers to *Acta Crystallographica C*. A number of extensions to this dictionary are in preparation. By storing the dictionary itself as a STAR file, the definitions and relationships in the CIF dictionary become computer interpretable. This offers many possibilities for the automatic handling of crystallographic information.

## 1. Need for a Crystallographic Information File

Crystallography is rich in numerical information. An x-ray or neutron diffraction pattern of a crystal typically consists of several thousand diffraction peaks, the intensities of which are used to determine the several hundred parameters needed to describe the positions and motions of the atoms. These coordinates are not themselves interesting, but they can be used to calculate the bonding geometry or to display the arrangement of the atoms on a screen. It is therefore convenient to keep the information in an electronically readable form and for this purpose we need a file structure. If the file structure is widely accepted by the community, the information describing the crystal can be readily passed from program to program or from laboratory to laboratory.

Traditionally the results of a scientific investigation are printed in a journal. A crystal structure determination requires that all the atomic coordinates be printed (and, in principle, also the diffraction amplitudes, since they are the primary measurements). The process by which the journal manually typesets extensive tables from a computer listing, and the reader of the journal subsequently keyboards the same numbers back into the computer, is very inefficient and error prone. Recognising this, the International Union of Crystallography (IUCr) decided in 1990 to accept structure reports for *Acta Crystallographica C* in an electronic form generated by the software used for the structure determination. The numerical values in this submission were to be computer checked for consistency and the paper typeset by computer, before the electronic file was passed on to the crystallographic databases for archiving. To facilitate this process the IUCr established the Crystallographic Information File (CIF) as a standard for the transmission of crystallographic data.

Because crystallography, and particularly information technology, are rapidly evolving, it is necessary that the CIF standard also be able to grow. It has to be flexible, allowing for extension as the need arises and, as far as possible, allowing the author to use a CIF to express any relevant crystallographic results in the most appropriate way. A CIF can be compared with an essay; both contain information expressed in a language in which rules of syntax link the lexical words whose meaning is defined in a dictionary. Both are also able to convey a wide range of information. The difference between them is that information in an essay is processed by our brains, while the information in a CIF is processed by a computer. This difference shapes the differences in the languages.

## 2. The Syntax of a Crystallographic Information File

The crystallographic information file uses the STAR file structure [1,2] whose basic syntax is quite simple. A STAR file consists of strings of characters (words) separated by one or more blanks. There are two kinds of string: data names, which mostly start with the underline character ‘_’; and data values, which start with any other character (except of course a blank which is used as a separator). In a STAR file each data item consists of a data name followed by its value:

**Table:** 

_cell_length_a	7.345
_cell_length_b	9.134
_cell_length_c	5.456

Loops are allowed and are introduced by the data name ‘loop_’. In this case the data names are all given at the beginning of the loop and the numerical values are assigned to each data name in turn, returning to the beginning of the list each time the list of names is exhausted. The loop ends when another data name is read:

**Table:** 

loop_			
_atom_site_label			
_atom_site_fract_x			
_atom_site_fract_y			
_atom_site_fract_z			
C1	0.4145	0.2460	0.25
C2	0.3284	0.1890	0.7892
C3	0.9389	0.7238	0.1256
_next_data_name		*#* terminates the loop	

This example shows that all the characters on a line following the character ‘*#*’ are comments and are not considered part of the information in the file. In the above examples the various data names and data values have been formatted so that they are easy to read. This is not necessary. The names and values can be written sequentially providing each string is separated by at least one blank or an end of line:

loop_ _atom_site_label _atom_site_fract_x _atom_site_fract_y atom_site_fract_z C1 0.4145 0.2460 0.25 C2 0.3284 0.1890 0.7892 C3 0.9389 0.7238 0.1256 _next_data_name

Strings that contain a blank must be surrounded by ’ ’ or ” ”:

_symmetry_space_group_name_H-M ’P n m a’

and strings that extend over more than one line have a semicolon ‘;’ as the first character on the first line and the first character on the line following the end of the string:

;This is an example of a piece of text that is so long that it requires more than one line to write it all down. The text can be as long as required. There is no limit to the number of lines, but the text field is terminated with a line that contains a semicolon, ‘;’ as the first and only character.

;

The information in a file is divided into blocks, each block containing a self-contained set of information, for example, the information needed to describe one crystal structure. Each block starts with the data name:

data_user-identifier

and ends with the next ‘data_’ statement or the end of file. In this way it is possible to store the results of several structure determinations in the same file.

This is the basic STAR syntax and the grammar of a CIF.

## 3. The CIF Dictionary

Before one can construct a CIF one needs, in addition to a grammar, a dictionary of data names, and this is where the structure starts to become interesting. A normal dictionary is designed to be read by people, but to understand the dictionary, one must first have a basic knowledge of the language since each word is defined in terms of other words.

The first CIF dictionary [3] was similar to an ordinary dictionary. The data names were defined in terms that a crystallographer would understand and, like a normal dictionary, sometimes included examples. In addition it gave information that was required to ensure that the computer program could read the CIF. The entry for the measured density is typical:

**Table:** 

**_exptl_crystal_density_meas**	(*numb*)
Density values measured using standard chemical and physical methods. The units are megagrams per cubic metre (grams per cubic centimetre). The permitted range is 0.0 → ∞	

This example shows the density to be defined as a positive number. This is necessary if the computer is to use it in a calculation, since a non-numeric ASCII string such as “The density was not measured,” would cause the program to fail. In general, all the computer can do with a non-numeric string is to store it and print it out. If the computer is expected to understand the meaning of an ASCII string, the string must have a precisely defined structure. An example is the Hermann-Mauguin space group name listed in Sec. 2. Because instructions for writing this symbol are given in the dictionary, the symbol can be parsed by a computer and used to generate a list of equivalent positions. The first dictionaries explained to the crystallographer how to construct the symbol, and to the programmer how to ensure that the computer could parse it, but they did not include definitions that the computer itself could read and interpret.

STAR files are not restricted to crystallography. They can store any kind of information such as information on astronomy, cooking or stamp collecting; all that is needed is a dictionary to define the various data items that need to be stored. Not surprisingly, there have been proposals for other dictionaries, and some of these are discussed below. Soon after the first version of the CIF dictionary had been prepared, it was suggested that the information in this dictionary could itself be stored as a STAR file. All that was needed was a dictionary that contained data names that were appropriate to the information stored in dictionaries, data names such as:

_name
_definition
_example

The first CIF dictionary was therefore stored as a STAR file. The entry for the measured density appears as a data block in this file as follows:

**Table:** 

data_exptl_crystal_density_meas	
_name	’_exptl_crystal_density_meas’
_category	exptl_crystal
_type	numb
_list	both
_list_reference	’_exptl_crystal_id’
_enumeration_range	0.0:
_definition	

; Density values measured using standard chemical and physical methods. The units are megagrams per cubic metre (grams per cubic centimetre).

;

This computer-stored dictionary was originally used to test the programs that were being written to convert STAR files into Tex for typesetting, but it soon became clear that storing the dictionary as a STAR file had implications that extended far beyond typesetting. It meant that the computer, as well as the programmer, could read the dictionary, and this implied that, as far as possible, all the data items defined in the dictionary ought to be made machine interpretable. In the example above, a computer can, by reading the dictionary, determine that the value stored following the name _exptl_crystal_density_meas must be a positive number. The information given under ‘_category’, ‘_list’ and ‘_list_reference’ tell it about the way the density is related to other information in the CIF.

It was always possible to write a program to check if a CIF complied with the STAR syntax, since the program could distinguish between data names (beginning with ‘_’) and data values, but the computer would treat the data names and data values as strings of characters with no particular meaning. By making the dictionary itself available as a STAR file, a program could be written to compare a CIF with its dictionary and check whether the data names used in the CIF were defined in the dictionary and whether the data values lay within the prescribed ranges. For example, it could check that the density is given as a positive number. Similarly, the Hermann-Mauguin symbol could also be checked for legitimacy, if the rules for its construction are given in a machine interpretable form in the dictionary. Further possibilities suggested themselves. The loops in a CIF can be regarded as tables, and pointers between tables can be defined, allowing a CIF to be mapped directly into a relational database. One can even imagine a day when the dictionary will contain the algebraic relationships between the different items, so that a program with no knowledge of crystallography could use the dictionary to discover how to calculate bond lengths from the atomic coordinates. We are still a long way from achieving this goal, but ensuring that the definitions in the CIF dictionary can be interpreted by a computer is a first step.

Current drafts of the CIF dictionary do not contain algebraic definitions in a machine-readable form, but they do contain the pointers needed to read a CIF into a relational database. Where the information in two loops (or tables) is related, e.g., coordinates and bond lengths, pointers are set up, identifying which entry in the coordinate table (i.e., which atom) is involved in a particular entry (i.e., which bond) in the table of bond lengths. The rules for establishing the contents of different tables and setting up the pointers are somewhat complex and are still being developed, so there is no point in elaborating them here. But structuring a CIF as a relational database allows for the possibility of direct mapping of the crystallographic information into a relational database that can use existing general software to explore relationships between structures.

Since the first CIF dictionary appeared several other dictionaries have been defined. There is now a dictionary for dictionaries—the Dictionary Definition Language (DDL) [4] and a proposal has been made for a second dictionary definition language (DDL2) with much tighter rules for defining the relationships between data items. At the crystallographic level, extensions to the CIF dictionary are being prepared to cover macromolecular structures, powder diffraction, modulated structures, symmetry and graphics. The *World Directory of Crystallographers* is stored as a STAR file with its own dictionary, there is a dictionary for a Molecular Information File (MIF) for use in chemistry, and a proposal for an information file containing the results of NMR studies of macromolecular structures.

Much of the impetus for the development of the CIF dictionary has come from the macromolecular community who are developing ways of using computers to look for relationships between the structure and properties of proteins and other biomolecules. Here the aim is to bring together information from different sources (protein and DNA sequences, x-ray diffraction, 2-D NMR). The flexibility of the STAR syntax makes it particularly suitable for this purpose.

## 4. Uses of Crystallographic Information Files

The IUCr adopted the CIF standard to ensure that the information on crystal structures could be archived without introducing the keyboarding errors that are inevitable in a system requiring that tables of coordinates be retyped at least three times, as was the case until recently.

Starting in January 1992, all papers submitted to *Acta Crystallographica C* have been typeset from CIFs. Initially this meant keyboarding the papers received in the editorial office into CIFs, but increasingly, as the software for creating CIFs has become more available, CIFs are generated automatically in the authors’ laboratories and are submitted electronically. When a CIF is received by the editorial office, it is immediately checked for syntactical correctness and the numerical information checked for consistency and plausibility. Only when these checks have been successfully passed is the paper sent for scientific editing. After any changes made at this stage, the CIF is corrected and used to typeset the paper. Finally, the CIF is made available to the appropriate crystal structure database. During the whole of this process, the numbers are never keyboarded; the numbers that go into the printed journal and database are the ones produced by the structure determination software.

The crystallographic community has adapted to the CIF technology remarkably swiftly; within 4 years of their introduction, CIFs are expected to be used for all submissions to *Acta Crystallographica C*. There are several reasons for this ready acceptance. Crystallographers pioneered the use of computers and most are comfortable with their use. The STAR syntax is relatively simple and CIF dictionaries are prepared by members of the crystallographic community who understand the meanings of the stored items and the uses to which they will be put. The willingness of the authors of structure determination software to provide an optional CIF output has simplified the writing of CIFs and, finally, the decision of *Acta Crystallographica C* to adopt the CIF technology as a package, rather than converting piecemeal, provided the necessary boost to persuade authors to learn the new approach. Having once mastered the art of submitting a paper as a CIF, most authors would not now want to return to the old method.

The crystal structure databases accept CIFs from *Acta Crystallographica* as this saves them keyboarding in newly published structures and reduces errors. Other journals have also agreed to accept CIFs for deposit with the databases in cases where they do not intend to print full details of the structure determination. Users of the databases will soon be able to download entries as CIFs. The Powder Diffraction File and the Protein Databank are considering using CIFs as their primary archive, and increasingly, public software that needs to input or output crystallographic information is providing a CIF option.

## 5. Advantages of the Crystallographic Information File

### 5.1 Flexibility

Some of the advantages of using CIFs have been discussed above, namely that the file structure is flexible and can grow with the discipline. New definitions can be added to the dictionary as the field develops. However, the old definitions can never be discarded since a program armed with the dictionary has to be able to read any file that conformed with the standard at the time it was written. This can lead to difficulties. A poorly defined data item cannot be replaced, though it is possible to introduce a new and better defined data item to perform the same job. The fear of loading the dictionary with obsolete data items inspires the dictionary writers to create definitions that will stand the test of time.

### 5.2 Simplicity of the File Structure

The structure of a CIF is simple, and the information it contains can be easily read from a listing. In the future even this will not be necessary as tools are developed to display the information in the CIF on a screen or to prompt the user for the information required. A crystallographer will need to know as much about the structure of a CIF as the user of a word processor knows about the structure of file the word processor writes. However, the CIF dictionary has a much more complex structure that makes reading a listing quite difficult. Fortunately, few crystallographers will have to consult the dictionary, only those writing programs, and they will have browsers to allow them easy access to the information they require.

### 5.3 Computer Compatibility of the Dictionary

The dictionary can be read by a computer which makes it possible for the computer to decide how to treat the information that it finds in a CIF. The information may be loaded directly into a relational database, individual items can be parsed according to rules contained in the dictionary and, in the future, relationships between data items will be included explicitly in the dictionary, leading to the possibility that the dictionary will contain all the information about crystallography that a computer will need to know.

### 5.4 Integrity of Information

The authors of a CIF must ensure that the data values in the file agree with the definitions in the dictionary. Because the computer cannot correct for lapses in human logic in the way that people can, a CIF is of little use if it does not conform to the exact dictionary definition. The computer will treat a poorly prepared file as nonsense ensuring that the results of any calculation are meaningless. Because they must conform to the standard, CIFs help to ensure the quality and integrity of the information they contain.

### 5.5 Definition of Crystallographic Terms

Even the exercise of preparing the dictionary focuses the attention of the community on the definitions of items that were previously only loosely defined and draws attention to relationships that had not previously been appreciated. Over the years a variety of R factors (agreement indices) have been adopted to express the agreement between the measured structure factors and those from a proposed model. Describing them in the dictionary requires a set of logical definitions and has drawn attention to the meaninglessness of terms such as wR(obs).

### 5.6 Easy Publication of Crystal Structure Determinations

For the person determining crystal structures, the CIF provides an easy route to publication. The structure-solving software writes all the numerical information, as well as details of the experiment, into a CIF. All the author has to do is add the title and a short text describing the source of the sample and a brief description of the results. The IUCr provides an automatic email prechecking service which returns an error report usually within an hour, allowing the CIF to be corrected prior to submission.

The work of the editorial office is greatly reduced. Nothing has to be keyboarded, the checking programs ensure the quality of the numerical information, papers can be processed more rapidly and typesetting is automatic. The databases, which are becoming the major archive for crystal structures, can assume that a CIF received from *Acta Crystallographica* is free of numeric errors and is ready for inclusion in the archive.

## 6. Administration of the Crystallographic Information File

The integrity of the CIF dictionary is important. The language of a CIF is much more brittle than ordinary language. Spoken and written language has rules that allow it to change. New needs in language can be met by inventing words or constructs whose meaning is apparent to the reader. The same is not true of CIFs. So far we have not developed rules which allow new data items to be defined automatically without ambiguity. The construction of the dictionary itself is still very much the task of crystallographers. Every data item must have a precise definition and a precisely defined structure which has to be encoded in the dictionary if a computer is to read and comprehend a CIF. For this reason, it is not feasible for users to invent their own data names and expect others to understand or adopt them. There has to be a central authority responsible for the definitions and structures of each new data item.

To prevent the development of CIF dialects, the IUCr has applied for a patent on the STAR file structure and has copyrighted the CIF dictionary. The intent is not to prevent their use, but to make sure that those who do use CIFs observe the standards established by the Union. To maintain and develop these standards, the IUCr has appointed a committee called COMCIFS, whose role is to review all proposals for extensions to the CIF dictionary, to ensure that they conform to the STAR syntax and do not violate any of the conventions of the current dictionary. Extensions that conform and are necessary for recording crystallographic information are then recommended to the IUCr for approval.

## 7. What Does the Future Hold?

In the future we shall see other uses of CIFs. They will be loaded into relational databases, allowing sophisticated searches to be performed with relative ease. We can expect the information in *International Tables for Crystallography* to be available as CIFs, and precoded graphical images will allow a user to start the exploration of a structure using a view that brings out its most important features. We are only slowly discovering all the possibilities that the STAR structure and computer based CIF dictionaries offer and it is difficult to predict all the things that CIFs will allow us to do. The reality is likely to outstrip anything that we can imagine at this time.

For further information contact the author or the Executive Secretary of the International Union of Crystallography, 2 Abbey Square, Chester CH1 2HU, UK (execsec@iucr.ac.uk). *A Guide to CIF for Authors* is available from the Editorial Office of *Acta Crystallographica*, 5 Abbey Square, Chester CH1 2HU, UK. (med@iucr.ac.uk; home page http://www.iucr.ac.uk/welcome.html).
